# Playing 7 vs. 6 with an empty goal: Is it really an option for coaches? A comparative analysis between Portugal and the other teams during the Men’s European Handball Championship 2020

**DOI:** 10.3389/fpsyg.2022.809909

**Published:** 2022-09-27

**Authors:** João Nunes Prudente, Americo Ramos Cardoso, Ana Jose Rodrigues, João Noite Mendes, Catarina Fernando, Helder Lopes, Duarte Filipe Sousa

**Affiliations:** ^1^Faculty of Social Sciences, University of Madeira, Funchal, Portugal; ^2^Escola Superior de Tecnologias e Gestão, University of Madeira, Funchal, Portugal

**Keywords:** handball, observational methodology, polar coordinate, attack, 7 vs. 6 with empty goal, patterns of play

## Abstract

The dynamic of changes in the rules in team games materialize from research and debate between experts and coaches before being implemented by the International Federations. In Handball, the last changes occurred in 2016, and one of them was to substitute the goalkeeper with an additional field player allowing teams to play “empty goal” while using the additional field player.

This study aimed to analyze and characterize the use of the 7 vs. 6 strategical-tactical option for the attack in the 2020 Men’s European Championship. We also analyzed whether the game time and partial score influenced the use of 7 vs. 6 and its efficiency. Observational methodology and a mixed *ad hoc* instrument combining field format and category systems validated by experts were used. Data were taken from 28 matches involving teams in the first 12 positions in the 2020 Men’s European Championship. The total number of offensive sequences in an organized attack method in 7 vs. 6 with an empty goal (*n* = 123) were analyzed. Sequential analysis techniques with lags, prospective and retrospective, and polar coordinate analysis were used. The results showed that (a) these solutions had poor efficiency, except for the Portuguese National Team; (b) teams using the 7 vs. 6 tactic option had no negative consequences or increased risk with the opponent response; (c) partial score influenced the decision to use 7 vs. 6 strategic option, and (d) game time was associated with 7 vs. 6 play options and detected patterns.

## Introduction

The changes to the rules of different sports aim to evolve through new realities and guide to a more engaging game. European and world sports organizations, using expert opinions, try to correct negative aspects or aspects that prevent a positive evolution of the game.

Handball is no exception. There was a need to improve the rule regarding goalkeeper participation in the offensive game. Efficient regulation of what was already accomplished by some coaches, the goalkeeper in the attack was replaced by a field player, or the goalkeeper was used as a field player. Thus, the rule “to substitute the goalkeeper with additional field player” was modify. It allows teams to play “empty goal” while using an additional field player (International Handball Federation, 2016).

The rule changes introduced in 2016 allowed their implementation during the main competitions since then. It has led to some studies conducted on this topic, namely, the 2016 Olympic Games (Krahenbühl et al., 2019b), European Men’s Championship 2018 (Korte and Lames, 2019), 2019 Women’s World Championship (Gümüs et al., 2020; Maroja et al., 2020; Spate, 2020), and Junior Men’s World Championship 2019 (Spate, 2019). Playing without Goalkeeper is also allowed in futsal where some studies have been conducted (Corrêa et al., 2014; Méndez-Domínguez et al., 2019).

As noted by Korte and Lames (2019), with this new rule, different attacking formations have increasingly occurred, such as 7 vs. 6 with two pivots, or 5 + 1 vs. 6, and teams are using this new offensive strategy mainly in situations of equality and numerical inferiority (Maroja et al., 2020).

According to Korte and Lames (2019) most attacks occurred in 6 vs. 6 (74.5%), 10.3% in 5 + 1 vs. 6 formation and only 3.9% in 7 vs. 6 formation. These authors observed that in the 5 + 1 vs. 6 formation, teams intend to lapse time while playing in numerical inferiority. The same behavior was observed when teams used 7 vs. 6, arguing that this behavior was motivated by being playing without goalkeeper and fearing to suffer a goal after a turnover, as Prudente et al. (2019) and Krahenbühl et al. (2019b).

The results (3.9%) obtained by Korte and Lames (2019) in the World Men’s Championship 2017 are different from the results observed by Prudente et al. (2019) in the same competition (11.5%) for 7 vs. 6 formation. It is important to refer that Prudente et al. (2019) considered only the four best ranked teams.

Marczinka and Gál (2018) also analyzed the World Men’s Championship 2017 and found that teams used empty goal tactic when they were in numerical inferiority (twice as often when they were in numerical superiority) and, considering the partial score, they used more often when losing than when wining.

Prudente et al. (2019) also studied competition to evaluate the influence of game situations with an “empty goal” on tactical behaviors in the attack.

Abbreviations: SF, No finalization; GD, Goal-to-goal attempt.

More recently, in the same competitions, Gümüş and Gencoglu (2020) studied the effects of the goalkeeper substitution rule as a new strategy in Handball. They concluded that teams that used this strategy had no extra efficiency in attack, but they noted that its use had no negative consequences or increased risk for teams playing with an empty goal.

It is essential to continue to deepen the study of the implications of this rule in the game. Not only is it not consensual among coaches (Krahenbühl et al., 2019a), but also, being a relatively recent change, there is a lack of studies on it. As these authors state, the possibility of attacking 7 vs. 6 with an additional player can transform a teams’ tactical behavior because causing spatial and temporal changes alters the strategic-tactical behavior due to the new conditions during the game.

A previous qualitative study (Krahenbühl et al., 2019a) that held interviews with elite coaches, who had not yet registered the significant strategic changes in Handball stressed the need for further studies on this topic over a more extended period to identify the possible changes to the game.

In the study by Maroja et al. (2020), the results showed that the efficiency of teams in a 6 vs. 6 attack formation with empty goal was greater than that obtained when attacking 7 vs. 6 (winners - 48.5% vs. 47.6%; losers - 40, 7% vs. 33.3%).

Flores-Rodríguez and Ramírez-Macías (2021) have analyzed the World Men’s Championship 2019 and referred that during this championship teams played with empty goal in three different situations: in numerical equality, to obtain numerical superiority in attack; in numerical inferiority, trying to compensate the excluded player; and, also in numerical superiority. In this study, the results point to a lower use of the empty goal in numerical equality and that the efficiency of these attacks is lower than when they played without an empty goal in numerical equality. They also observed that using the empty goal tactic in a situation of numerical equality harmed defensive retreat.

All high-level competitions in Handball provide an opportunity to observe and analyze the evolution of the game and assess the introduced changes in the rules. Men’s European Handball Championship, the Olympic Games, and the Men’s World Handball Championship are, currently the three highest-level competitions. Since the rule of empty goal with the additional field player was introduced in 2016 at the Olympic Games, this European Championship presented an opportunity to evaluate this change and perceive how coaches and teams manage this strategical-tactical possibility during the competition after the 2017 Men’s World Championship, when the number of 7 vs. 6 attacks with empty goals was poor (Prudente et al., 2019). There were 19 attacks in numerical superiority without a goalkeeper (empty goal) playing 7 vs. 6, 25 attacks in numerical inferiority, and 106 attacks in numerical equality. Two years later, 4 years after the implementation of the new rule, we ask: Considering the utilization of this possibility, are there novel approaches by teams and coaches during the game?

It is important to recognize the improvement of the 7 vs. 6 attack numbers. This attack option passed from 19 situations in 2017 to 123 in 2020; hence the need to study and observe current trends.

Anguera and Hernández-Mendo (2013, 2014) refer an increasing interest from the sport researchers in the observational methodology in the last decade, due to its flexibility, accuracy, precision, and ability to be used for complex situations, such as team sports. According to them this methodology has now been used in relation to different sports such as soccer, basketball, handball, tennis, swimming, athletics, judo, and polo.

Several studies on handball have used the observational methodology and the analytical techniques of sequential analysis with lags, and polar coordinates, which proves its validity for studies in handball (González et al., 2013; Morillo et al., 2015; Sousa et al., 2015; Flores-Rodríguez and Anguera, 2018; Navarro et al., 2018; Prudente et al., 2019; Jiménez-Salas et al., 2020; Maroja et al., 2020; Quiñones et al., 2020).

Our goal was to analyze, characterize, and evaluate 7 vs. 6 and its use during the Men’s European Championship 2020. As Portugal National Team used the most 7 vs. 6 strategic-tactical organization (Portugal - 55% vs. all other teams - 45%), we wanted to compare Portugal’s results with the other 11 ranked teams. In addition, we aimed to characterize and analyze the influence of playing time and the partial result in the tactical use of the 7 vs. 6, its effectiveness, and shooting zones.

## Materials and methods

The study used an observational design, idiographic (all the sequences 7 vs. 6 were observed as a unit in the same competition), multidimensional (several response levels were studied), follow-up (several games played in the same championship were observed), located in the first quadrant and type 1 data (Anguera, 2003; Anguera et al., 2011).

### Sample

A total number of offensive sequences (*n* = 123) were carried out in an organized attack game method and in 7 vs. 6 formation, were gathered from 28 matches involving the teams classified in the first 12 positions in the Men’s European Championship 2020). All 123 attacks occur in numerical superiority 7 vs. 6 without a goalkeeper (empty goal). Only the sequences that started and ended in 7 vs. 6 formation were considered.

### Procedures

We observe and collected data using a mixed *ad hoc* instrument consisting of a field format with category systems that was built and duly validated by 17 experts through a questionnaire, using a 5-point Likert scale (from 1-I Totally Disagree to 5-I Totally Agree) and to validate the categories, a cut-off value of 75% was considered. All coaches had at least 10 years of experience: seven had national competition experience, two were national team head coaches, and the rest had regional experience.

All offensive sequences were observed for each game. The first step was to exclude all sequences without an organized attack. The second step was to exclude all sequences of organized attack with empty goal in which the numerical relation was different from 7 vs. 6.

Our observation unit was the offensive sequence defined from the moment the team started the attack in a 7 vs. 6 formation until the end of the opponents` team response. The observation ended with a fast break, fast attack by attempting a goal-to-goal shot, or for an organized attack situation after the throw-off. Another situation was when the 7 vs. 6 situations ended with 2 min exclusion for one of the teams. When a throw-in or timeout was made, it was considered a new sequence.

The videos of the games were obtained from EHF TV broadcasts and directly recorded on a MacBook Air 13″ computer, 2017.

Exclusion criteria were established: all periods that were more than 10% unobservable (Anguera, 1990) were eliminated.

### Instrument

The instrument used to collect data was a mixed *ad hoc* instrument consisting of a field format with category systems, with 11 criteria and 77 categories (Table 1).

**Table 1:** Observation instrument.

Criteria	Description	Categories description	Codes
Teams	Teams according to their order at the competition final table	1º position 2º position 3ºposition 4ºposition 5ºposition 6º position 7º position 8º position 9º position 10º position 11º position 12º position	T1 T2 T3 T4 T5 T6 T7 T8 T9 T10 T11 T12
Game time	Periods of game time	0´–10´ min of play 10´01´´- 20´ 20´01´´- 30´ 30´01´´- 40´ 40´01´´- 50´ 50´01 - 55´ 55’01”- 60’ 60”01”-65’ 65’01” – 70’ 70’01” – 75’ 75’01” – 80’	A1 A2 A3 B1 B2 B3 B4 P1 P2 P3 P4
The partial score	The result on the moment that begin the 7 vs. 6 situation	Draw Victory by one Victory by two Victory by three Victory by four or more Defeat by one Defeat by two Defeat by three Defeat by four or more	E V1 V2 V3 V4 D1 D2 D3 D4
Defensive system	Organized defence	Defence zone 6:0 Defence zone 5:1 Defence zone 3:2:1 Defence zone 3:3 Defence zone 4:2 Defence mixte 5+1 Defence mixte 4+2 Individual defence	6_0 5_1 3_2_1 3_3 4_2 5+1 4+2 H_H
Offensive organization	Organized attack related with number of pivot players	Attack with one pivot player Attack with two pivots players	1Pv 2Pv
Tactical means	Tactical means used during attack 7 vs. 6	Individual tactic means (shot, feint) Group tactical means (cross, position change block, screen, fly)	Ind Grp
Shot	Kind of shot used to score	9 m shot 6 m shot Wing shot Pivot shot Breakthrough shot No shot	R1L R6m RPt RPv RPn SR
Attack result	Result after shot to the goal	Goal No goal 7 m with goal 7 m no goal No shot by technical fault No shot by opponent action	G NG 7MG 7MNG SFFt SFAa
Zone	The shot zone of the offensive sequence	Z1 – Left wing attack Z2 – 6 mts – line player Z3 – Right Wing attack Z4 – Left back from 9 to 15 m Z5 – Centre Back from 9 to 15 m Z6 – Right back from 9 to 15 m Z7 – Left From 15 to 20 m Z8 – Centre From 15 to 20 m Z9 – Right from 15 to 20 m Z10 – Defensive area including GK area 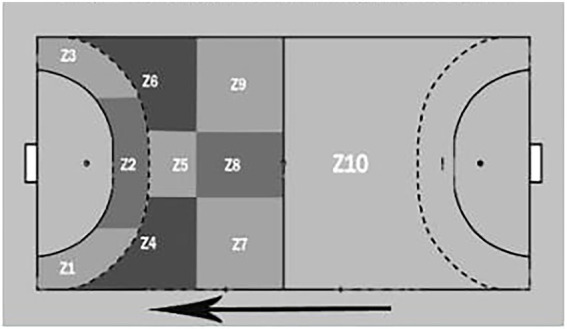	Z1 Z2 Z3 Z4 Z5 Z6 Z7 Z8 Z9 Z10
Opponent response	Opposing team’s response after regaining possession of the ball	Goal-to-goal attempt Direct fast break Sustained fast break Throw-off Fast attack Organized attack No response	GD CAD CAA Rep AR AO NE
Opponent response result	Result of the opposing team’s response	Goal when the opponent response finished scoring a goal No goal when the opponent response finished with a shot but no scoring a goal No goal with penalty No goal with opponent action	Golo NGolo NGcP SA

Data were observed and recorded using Lince software v.1.2.1. (Gabin et al., 2012). SDIS-GSEQ v. 5.1.23 (Bakeman and Quera, 1995), and software Hoisan v. 1.6.3.3.5 (Hernández-Mendo et al., 2012; Rodríguez-Medina et al., 2019) were used to analyze the data.

### Quality of data

Data and observer reliability were assessed using Cohen’s Kappa (Cohen, 1960) to assess intra-and inter-observer agreement.

Anguera et al. (2000) promote a preparation period to achieve observers’ reliability. In our study, the observers’ reliability was tested after a period of preparation and observation training using this instrument and after every six sessions, and in the last session of observation. The observers are handball coaches, with training and experience, having participated in different studies using observational methodology. During the preparation period, they observed four games. At the end of each game, they gathered and discussed the differences detected to standardize criteria in the registration. After this training period, each of the observers performed twice the observation and registration of same game. Between the two observation sessions, each observer respected an interv of 2 days. Kappa test was performed to verify intra and interobserver reliability.

Randolph (2005) suggests a 0.70 or higher to ensure reliability. The values obtained (intra-observer between 0.79 and 0.91 and interobserver between 0.87 and 0.94) determined the data quality and the observers’ reliability.

### Statistics

For statistics, GSEQ software was used for descriptive analysis (absolute and relative frequencies) and the sequential analysis technique with lags (Bakeman and Quera, 1995, 2011). Sequential analysis with lags is a probabilistic process that allows knowing the structure of the behavioral flow, from a given behavior which are explainable by more than chance (Bakeman and Gottman, 1986; Anguera, 1990; Hernández-Mendo and Anguera, 2002; Prudente et al., 2010).

Hoisan software was used to analyze the data using the polar coordinate technique. This technique reduces the amount of information, is more consistent with lower frequencies, and has been used in other studies for Handball (Sousa et al., 2015; Prudente et al., 2017; Flores-Rodríguez and Anguera, 2018; Jiménez-Salas et al., 2020).

The polar coordinates technique applies sequential analysis with lags, both prospective and retrospective, and gives a vector map with the relations between conducts (Hernández-Mendo, 1999). This technique allows locating the vectors in the quadrants and determining their angles, thus establishing the type of relationship between the focal conduct (given) and the conditioned conducts defined for each analysis. The length of the vector expresses the quantitative relationship between the focal conduct (given) and the conditioned conducts (the longer the vector, the stronger the intensity of the relationship between the conducts). The quadrant where the vector is placed expresses the qualitative relationship between these behaviors as follows (Paulis and Hernández-Mendo, 2003; Flores-Rodríguez and Anguera, 2018; Pastrana-Brincones et al., 2021):

Quadrant I (+ +). Mutually excitatory given conduct and matching conduct.

Quadrant II (−+). Inhibitory given conduct and excitatory matching conduct.

Quadrant III (−–). Mutually inhibitory given conduct and matching conduct.

Quadrant IV (+ −). Excitatory given Conduct and inhibitory matching conduct.

## Results

### Descriptive analysis

A descriptive analysis of data was carried out (absolute and relative frequencies) to make decisions for the following analysis both sequential analysis with lags and polar coordinate analysis (Anguera, 1986).

There were 1,228 events registered of 123 offensive sequences in 7 vs. 6 situations (empty goal) carried out by the first 12 teams in 28 games of the Men’s European 2020 Championship.

**Figure 1:**
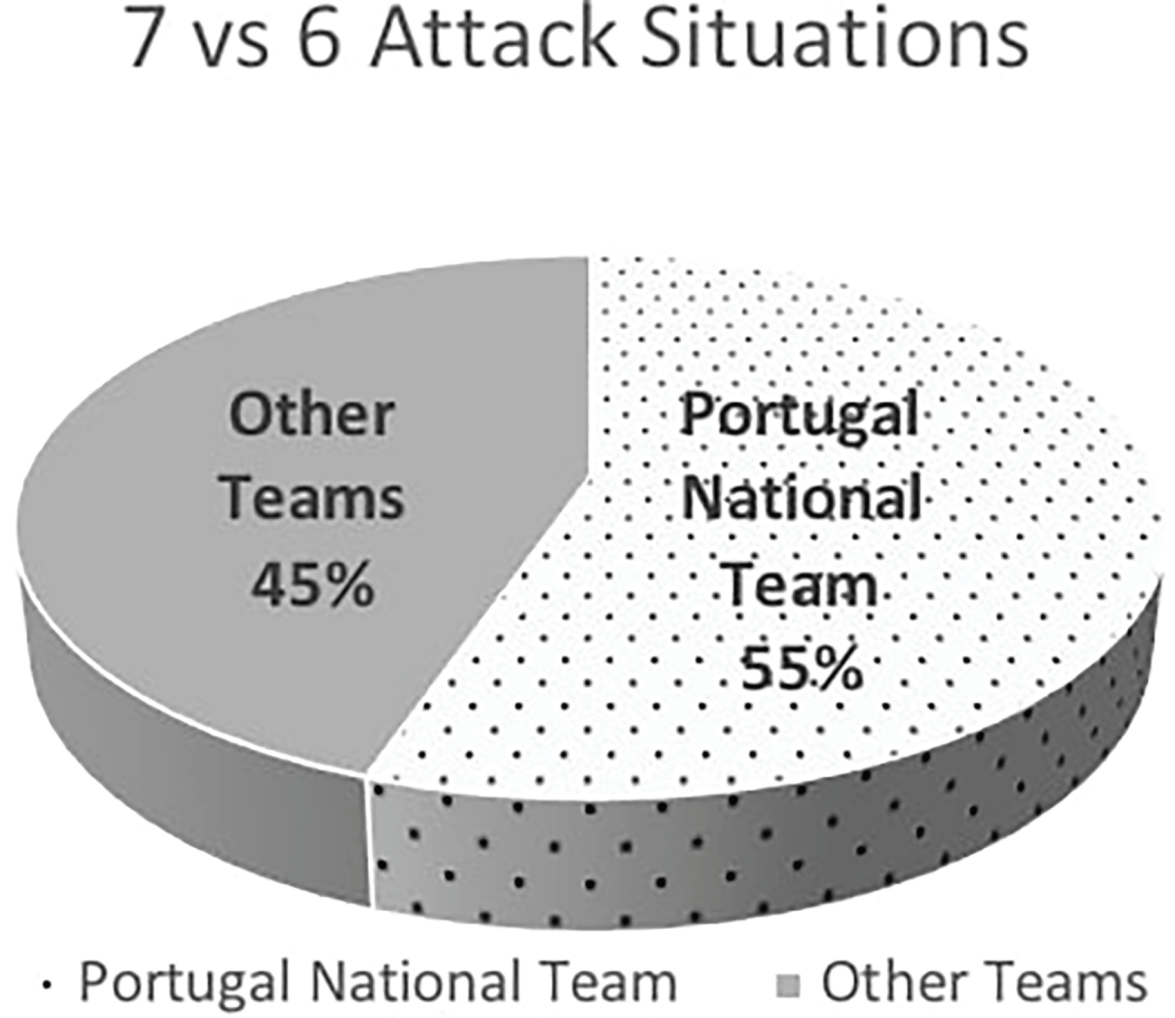
7 vs. 6 attack situations (empty goal).

The results show (Figure 1) that Portugal opted to play 7 vs. 6 in an organized attack 68 times (55% of all occurrences), and all other teams only 55 times (45% of all occurrences), but of these 11 teams, only Croatia (25), Sweden (24), Austria (3), Norway (2) and Hungary (1) showed results.

To understand why the teams applied this offensive organization, considering the partial result, we defined the results as balanced (Victory V123 - considering V1, V2, V3; Defeat D123 - considering D1, D2, D3) and unbalanced results (Victory - V4; Defeat - D4). This yields more significant values.

**Figure 2:**
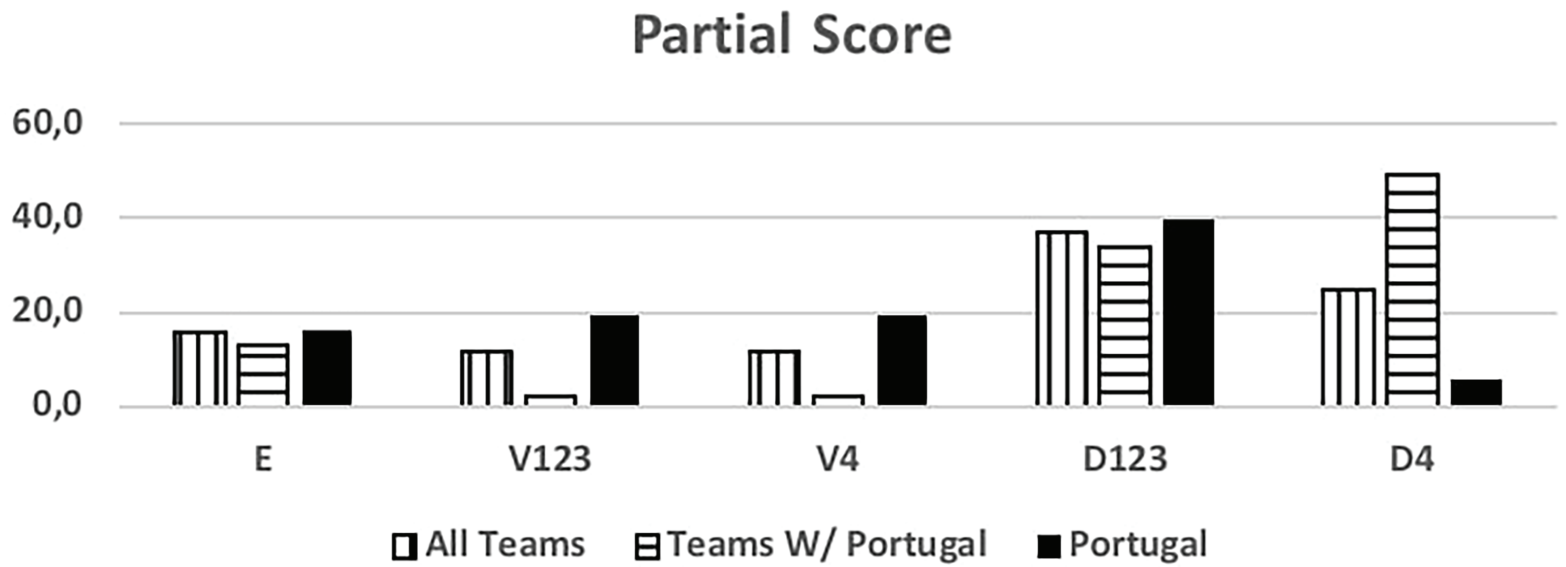
Partial score. E, draw; V123, winning by one, two or three goals; V4, winning by four or more goals; D123, defeat by one, two or three goals; D4, defeat by four or more goals.

Figure 2 illustrates how teams showed a preference for using 7 vs. 6 offensive situations when the result was negative while facing a Defeat situation.

**Figure 3:**
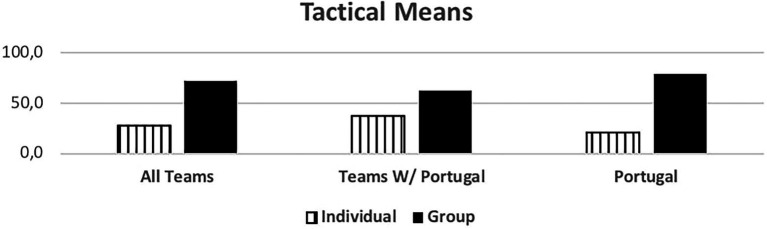
Tactical means. Individual tactical means – Shot, feint; Group tactical means – cross, position change, block, screen.

Figure 3 shows which response between the individual or group (two or three players) tactical mean is used more often. Most teams supported their actions through a collective response.

The results also indicate that the Portuguese National Team used group tactical means more often, primarily in two against two situations, such as blocks and crosses.

Excluding them from the analysis, increases the individual tactical means rate among the other teams.

**Figure 4:**
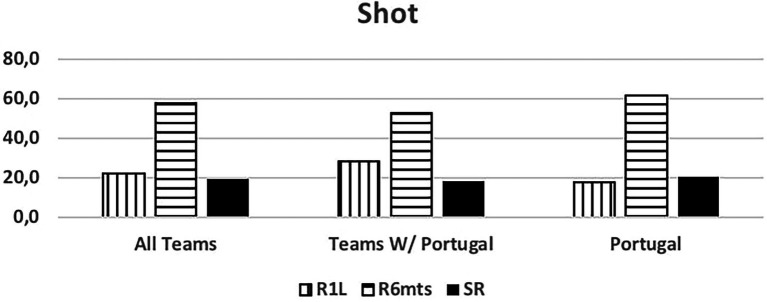
Shot. R1L, 9 m shot; R6mts, 6 m shot; SR, no shot.

Figure 4 analyzes the type of shot. We observed that the 6 m shot was most common, probably because most teams played with two wings and two pivot players.

Analyzing the Portuguese National Team results indicated that 9 m shots occurred less often than the other teams.

We codified efficiency “Efic” for all scoring situations and 7 m shots regardless of whether the 7 m shot led to a goal. We codified the process of the information and data in a meaningful way.

**Figure 5:**
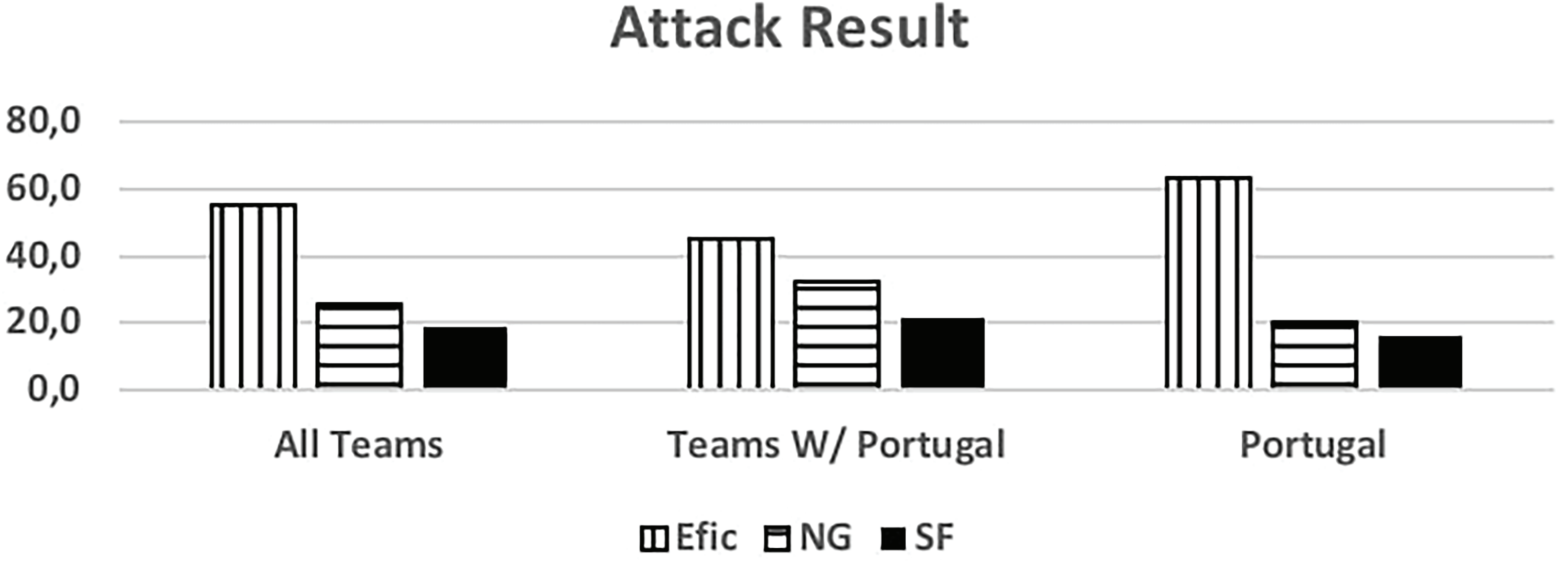
Attack result. Efic, efficiency (goal, 7 m goal, 7 m no goal); NG, no goal; SF, without shot.

Figure 5 verifies that the Portuguese National Team presents superior results regarding efficiency. This may indicate that Portugal team takes advantage of the attacking scenarios playing with offensive organization 7 vs. 6.

**Figure 6:**
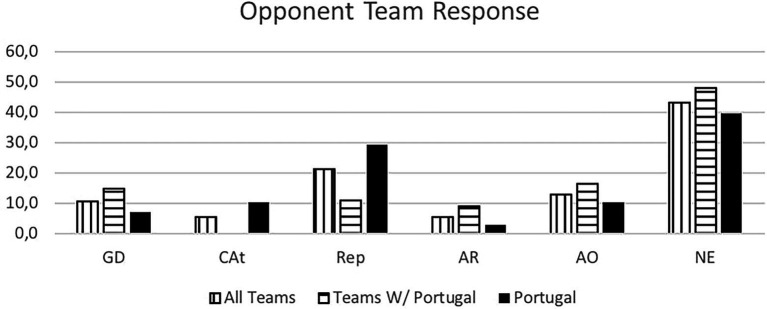
Opponent team response. GD, goal-to-goal attempt (when the goalkeeper or a defensive player tries to shoot from the Goal or from the defensive side of the field); CAt, fast break; Rep, throw-off; AR, fast attack; AO, organized attack; NE, no attack (teams abdicated of fast response).

We can also observe in Figure 6 that excluding the Portugal team the results present a higher no goal percentage.

We also examined of the response that the opposite team deployed more often after a 7 vs. 6 attack.

There is a mixed answer between throw-off, goal-to-goal attempt, and slow ball reposition. The slow ball reposition would allow the Goalkeeper entrance on the team attacking an empty goal situation.

However, it should be noted that the quick throw-off was most used by the opponents of the Portugal National Team.

**Figure 7:**
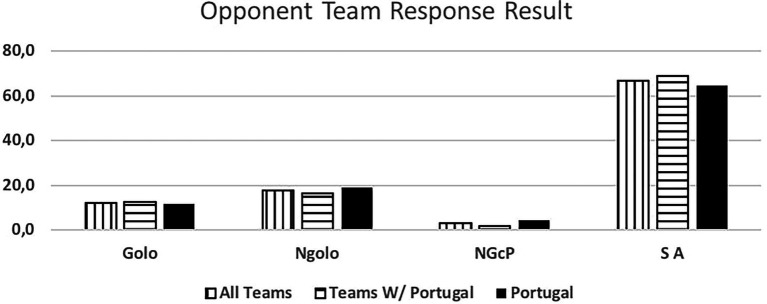
Opponent team response. Result golo, goal; Ngolo, no goal; NGcP, no goal with penalty (yellow card, 2 min, red card); SA, no response.

Figure 7 approaches the “Opponent Team Response Result.” We observe that the response often leads to a “No Goal” outcome than a “Goal” outcome.

### Sequential analysis

Sequential analysis with lags allows us to assess the probability of a relationship between conducts beyond chance. It consists in determining how the occurrence probabilities of certain conducts vary in relation to the prior occurrence of others (Hernández-Mendo and Anguera, 2002).

Based on the Focal Conduct (Given) “Shot” and conditioned conducts (Targets), the “Attack Result” and “Opponent Response,” this prospective analysis revealed different patterns of conduct associations considering Portugal National Team or all National Teams involved (including Portugal).

**Figure 8:**
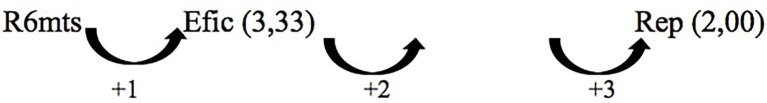
Association pattern between the focal conduct shot “R6mts” and the conditioned conduct attack result “Efic” and response of opponent “Rep” in Portugal National Team. R6mts, 6 m shot; Efic, goal or 7 m; Rep, throw-off.

The pattern detected with Portugal National Team reveals an excitatory association between “R6mts” (6 m shot) and the “Efic” (efficiency – Goal or 7 m) in lag one (3.33) and the “Throw-off” in lag three (2.00) (Figure 8).

**Figure 9:**
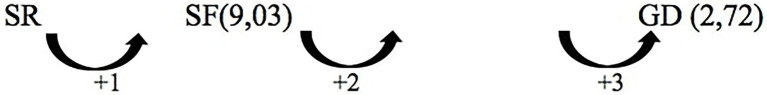
Association pattern between the focal conduct shot “R6mts” and the conditioned conduct attack result. “Efic” and opponent response “Rep” in all national teams. SR, no shot; SF, no finalization; GD, goal to goal attempt.

When the sample used was “All teams,” the pattern changed. As shown in Figure 9, starting from the same focal conduct “Shot” and the same conditioned Conducts “Attack Result” and “Opponent Response,” the SR conduct (No Shot) is associated with ineffectiveness “SF” (No Finalization) in lag one (9.03) and to the GD goal-to-goal attempt in lag three (2.72).

To understand if the game time conditioned or changed the relationship between the conducts, we carried out the same analysis but considering the influence of the Independent Variable “Game time.”

**Figure 10:**
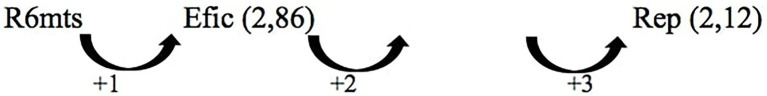
Association pattern between the focal conduct shot “R6mts” and the conditioned conduct attack result. “Efic” and opponent response “Rep,” considering game time factor/variable, in all national teams. R6mts, 6 m shot; Efic, goal or 7 m; Rep, throw-off.

**Figure 11:**
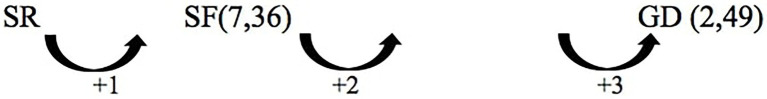
Association pattern between the focal conduct shot “R6mts” and the conditioned conduct attack result. “Efic” and opponent response “Rep,” considering game time factor/variable, in all national teams. SR, no shot; SF, no finalization; GD, goal-to-goal attempt.

This analysis shows that game time influences and changes the patterns, as can be seen in the comparison of Figures 9–11.

This situation confirms the appearance of a pattern that does not exist without the game time independent variable.

We also consider the relationship between the attack result, the opponent’s response, and the opponent’s response result. The association between conduct was found, which suggests the probability of situations in which there is no finalization (SF) in lag one (7.36) to be positively associated with the Goal-to-Goal Attempt (GD) in lag three (2.49) and the success of this attempt.

### Polar coordinate analysis

In this analysis, we used “All Teams,” including Portugal, as focal conduct (given) and observed how it conditioned the different categories: Tactical means (individual or group), Shot (9 m shot, 6 m shot, no shot), and the attack result (efficiency: Goal, 7 m with goal, 7 m without goal, no goal, and no shot).

**Figure 12:**
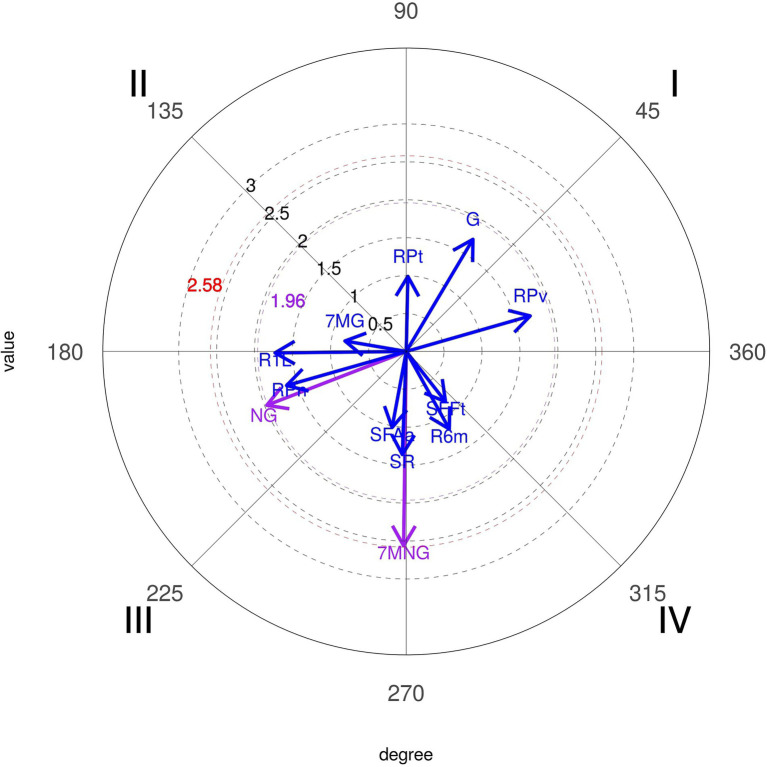
Polar coordinate analysis map: focal conduct “All National Teams.” Conditioned conducts: NG, no goal; 7MNG, 7 m no goal.

As we can see in Figure 12, the mutual inhibition relation of the given conduct (given) and “No Goal” (1.97) are significant. We also observed another significant result that, by relating the same conduct with the attack result, the probabilities of activation of the “7 m without goal” (2.56) were significant.

**Figure 13:**
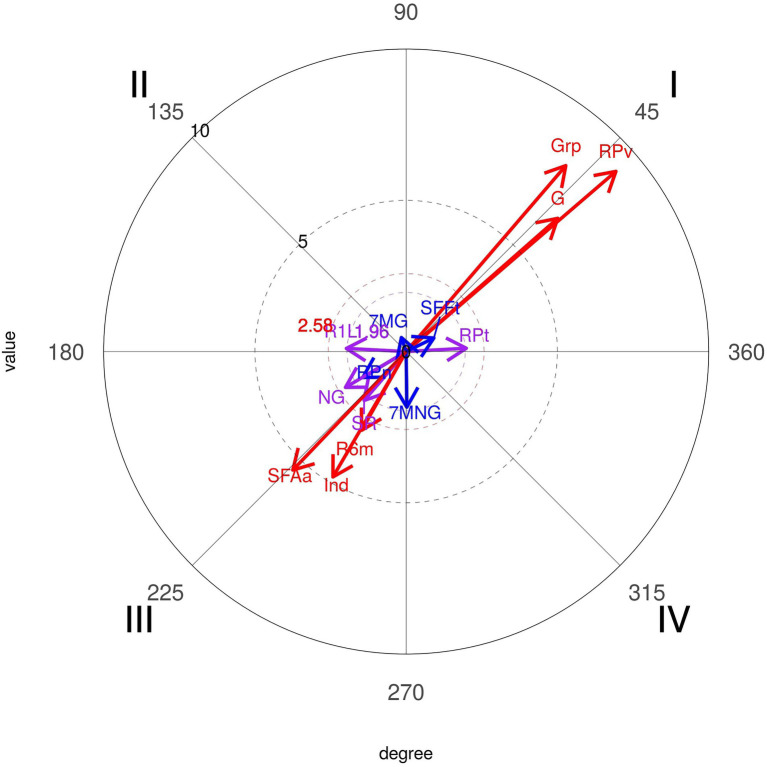
Polar coordinate analysis map: focal conduct “Portugal National Team.” Conditioned conducts: Grp, group tactical mean; Ind, individual tactical mean; RPv, pivot shot; RPt, wing shot; R1L, 9 m shot; R6m, 6 m shot; SR, no shot; G, goal; NG, no goal; SFAa, no shot by opponent action.

Comparing these results to the Portugal National Team (Figure 13), we can observe different relationships. These results showed significant associations with positive results (Figure 13). There is a significant probability that the Portuguese National Team is associated with group tactical means (8.1), pivot (9.14), and wing shot (1.99), and the most important association is with Goal (6.67).

On the contrast, they also show mutual inhibition with the Individual Tactical Means (4.8), No Shot (2.13), No Shot by Opponent action (5.42), and most importantly, “No Goal” (2.33).

**Figure 14:**
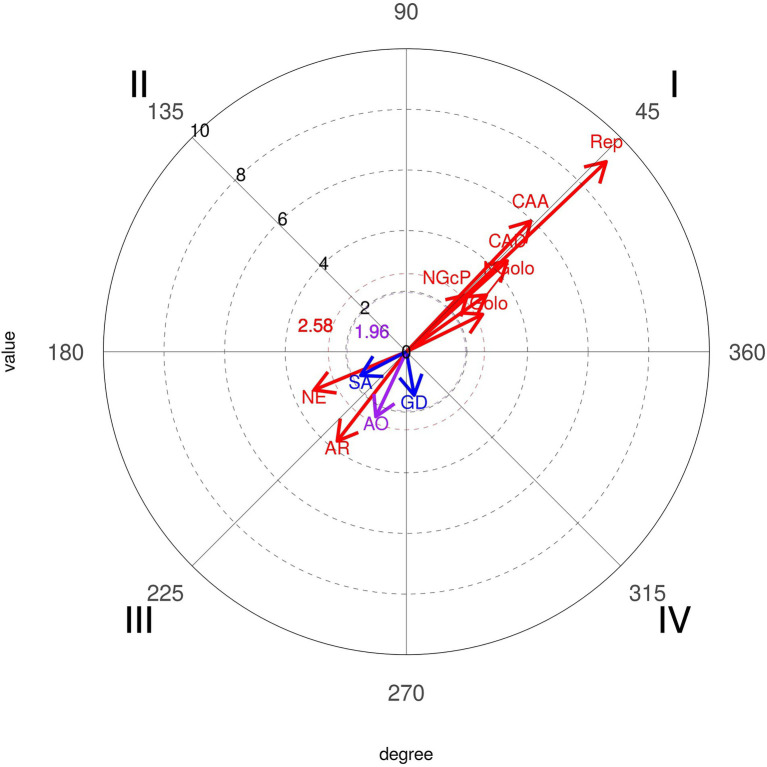
Polar coordinate analysis map: focal conduct “Portugal National Team.” Conditioned conducts: Rep, throw-off; AR, fast attack; NE, no response; AO, organized attack; CAD, direct fast break; CAA, sustained fast break; NGolo, opponent no goal; Golo, opponent goal; NGcP, no goal with penalty.

When the given conduct corresponds to the “Portugal National Team,” the conditioned conduct refers to the opponent’s response and the result to the opponent response. The results obtained are shown in Figure 14.

Significant findings were obtained for this relationship. Considering the Portugal National Team, a significant probability of inhibition of “Organized Attack” (2.37), Fast Attack (3.73), and No Opponent Response (3.3) were observed.

Furthermore, it was also established that the probabilities of activating the “Throw-off” (9.1), “Direct Fast Break” (4.48), “Sustained Fast Break” (5.95), “No Goal with Penalty” (2.76), “Goal” (2.8), and “No Goal” (3.24) were significant. It is important to note that even though the result of the opponent’s response “Goal” is activated, it is not associated with the attempt of a direct shot from goal-to-goal.

Considering “All Teams,” results showed that only the mutual activation relationship of the given conduct and “Throw-off” (2.59) was significant. Mutual inhibitory relationships were also detected with the “No Action” (2.03).

## Discussion

This study aimed to analyze and characterize the use of the 7 vs. 6 strategical-tactical options in the attack and to compare Portugal National team with the other teams, in the 2020 Men’s European Championship, because as results show Portugal National team was the team that most explored this tactical option (55% of the total sample). It was also analyzed whether the partial score and game time influenced the use of the 7 vs.6 tactic option. Our data suggests changes in the game compared to the 2017 Men’s World Championship. One of the differences was the number of 7 vs. 6 situations that occurred: there were 19 occurrences in a 12 games sample of the first four ranked teams, in 2017 (Prudente et al., 2019); in the 2020 competition, there were 123 occurrences in 28 game samples of the first 12 ranked teams. Croatia, second place, had used this tactic 25 times, Norway, third, two times, Portugal, sixth, 68 times and Sweden, seventh, 24. Spain National Team, first place as well as those classified in 10th, 11th, and 12th places did not use the empty goal during the main round and final phase.

We can suppose an increasing preference for the use of 7 vs. 6 tactic option but with different influence in its use by the different teams. Sousa et al. (2021b) showed that 58% of the coaches were in favor to change this rule as it is today, but even though some coaches are against this rule, others have adapted to it and prepared their teams to play 7 vs. 6 successfully, mainly, in some specific moments and situations, in order to play in numerical superiority (Marczinka and Gál, 2018; Krahenbühl et al., 2019b; Spate, 2020; Flores-Rodríguez and Ramírez-Macías, 2021). Sousa et al. (2021b) refer that from a sample of 125 coaches 76% agrees with the possibility to play 7 vs. 6. Gümüş and Gencoglu (2020) concluded that teams using this strategy had no extra efficiency in attack such as Trejo-Silva and Bonjour et al. (2021). Our data showed that the Portugal National Team was the pioneer of national teams to extensively use this option successfully, regardless of the result: Portugal used 7 vs. 6 regardless of the result (Winning - 19.1%, losing by three or less - 39.7% or tied - 16.1%, more than the other teams that used almost exclusively when they were losing in balanced games-34.1% or unbalanced games - 49.1% and only 1.9% when winning). This study also showed that partial results influenced the team behavior. In other words, most teams’ use the 7 vs. 6, more or less often depending on whether they were at a disadvantage or closer to the end of the game, as a desperate attempt for change as registered by other authors (Gümüş and Gencoglu, 2020; Maroja et al., 2020; Flores-Rodríguez and Ramírez-Macías, 2021). The results obtained by Marczinka and Gál (2018) are in line with this trend: teams used 7 vs. 6 tactic option twice as often as when they are wining. On the contrary, the Portugal team used 7 vs. 6 in different situations, either winning or losing, either in the first half or second, confirming it as a strategical-tactical option. In fact, the Portugal and Sweden National teams played 7 vs. 6 in the first half (Portugal - 15 times and Sweden-1) and in second half more teams used this tactical option: Portugal (53), Croatia (23), Sweden (24) and, Austria (3). During overtime, residual results were observed (5).

The Portuguese National Team was responsible for 55% of the total occurrences observed. Unanimously referred to as the 7 vs. 6 best team, we saw that some success was due to group tactical means (Portugal - 79.4% of attacks, other teams 63%) leading to 6 m shot (Portugal - 61.8%, other teams - 53.1%) and giving time to the goalkeeper replacement. Portugal was the best-ranked team, with over 60% success attacking this way (Portugal - 63.2%, and other teams - 45.5%), a higher score than the results (42.4%) observed by Trejo-Silva and Bonjour et al. (2021) related to World Men’s Championship 2017 and 2019, and European Men’ Championship 2018 and 2020. In our study the other teams supported 7 vs. 6 with more individual options (37% against 20.6% from Portugal), resulting in more errors and giving opponent teams success in the response. Compared to the Portugal team, their success rate was below 50%. Bonjour et al. (2021) state that in their study, the teams were able to retreat defensively and organize in defence. However, there was a considerable number of goal-to-goal attempts (15.7%) with an efficiency of 57%. According to these authors, this high number of goal-to- goal attempts is related to the ineffectiveness of the attack. As Gümüş and Gencoglu (2020) refer, the more missed shots and technical errors lead to more received goals by fast break or goal-to-goal attempt. It is important to note that playing with this system, the Portugal team had a positive balance between the opponents’ team response and its result. Portugal had half of the goal-to-goal attempt comparing to the other teams (7.4% vs. 14.8%). Many coaches had the perspective of a negative balance and a high number of goal-to-goal attempts. As the results show, the Portugal team had the lowest attempts of those shots from the opponents’ teams, contradicting some of the coaches’ opinions (Krahenbühl et al., 2019a; Iusepolsky et al., 2022). According to the authors, there were no significant changes occurred in handball neither in attack nor in defense with this new rule. In agreement with the results presented by the Portugal team, it should be noted that Sousa et al. (2021a) found that 86% of the Portuguese coaches surveyed agree with the possibility of playing 7 vs. 6 and 75% reinforce that this rule should be maintained, maybe influenced by National team results.

This study identified significant relationships between given conduct and conditioned behaviors. We identified a positive association between the given conduct 6 m shot with goal or 7 m in lag 1 (3.33) and throw-off in lag three (2.00) for Portugal National Team, which means that option to play 7 vs. 6, gave a chance to Portugal to shoot from nearer of the goal and therefore the probability to score a goal is significant. On contrary, the pattern detected for the other teams concerning this association show a significant probability of the no shot being associated to the goal-to-goal attempt (2.72) in lag three. These results prove that the Portugal National Team was effective playing 7 vs.6 in the attack, not conceding goals in the goal-to-goal attempts, while the other teams were not able to be effective being significant the probability to concede goals with goal-to-goal attempt. However, when we considered the game time as a factor, the pattern obtained for the other national teams changed and we can say that 6 m shot is positively associated with goal or 7 m in lag 1 (2.86) and with throw-off in lag three (2.12). The reason of this finding is that while the Portugal National Team used 7 vs.6 tactic option through all the game, the other teams only in have used in second half.

The polar coordinates technique reinforced the different applications and successes of the Portuguese National Team and others. It was significant that the probability of 7 vs. 6 activates success with Goal (6.67) and shots closer to the goal (Pivot-9.14 and Wing Shot-1.99) to Portugal National Team, in first quadrant, mutually excitatory, different to the other teams that had a significant probability of inhibiting 7 m with no goal (2.56), in third quadrant mutually inhibitory. Looking further into the results of the Portuguese team, we can see a significant probability that playing 7 vs. 6 inhibits goals from the opposite team (3.24), in first quadrant, mutually excitatory, confirming the results of Gümüş and Gencoglu (2020), who concluded that the use of this tactic option had no negative consequences or increased risk for teams playing with an empty goal when they scored a goal in attack.

## Conclusion

Our analysis allowed us to describe the existing association between 7 vs. 6 and a regular association with success and the impact of game time or partial score when using this strategy. We also identified behavioral differences between the observed teams, namely between the Portugal National Team and all the other teams observed. These results reflect significantly on this association. We believe it is important to continue studying this subject to understand the changes in the game better. Increasing the sample and observing more competitions are recommended.

The conclusions obtained can be extrapolated for 7 vs. 6 practice and its use in competition, not only in the last few minutes but throughout the game.

## Data availability statement

The original contributions presented in the study are included in the article/supplementary material, further inquiries can be directed to the corresponding author.

## Ethics statement

All procedures performed in studies involving human participants were in accordance with the ethical standards of the institutional and/or national research committee and with the 1964 Helsinki declaration and its latter amendments or comparable ethical standards.

## Author contributions

JP and DS developed the project, reviewed the literature, and wrote the manuscript. AR was responsible for performed analysis, the method section, and revised the content critically. AC, JP, and DS collected and analyzed the data and supervised the drafting of the manuscript. JM translated the manuscript. HL and CF revised the content critically. All authors contributed to the article and approved the submitted version.

## Conflict of interest

The authors declare that the research was conducted in the absence of any commercial or financial relationships that could be construed as a potential conflict of interest.

## Publisher’s note

All claims expressed in this article are solely those of the authors and do not necessarily represent those of their affiliated organizations, or those of the publisher, the editors and the reviewers. Any product that may be evaluated in this article, or claim that may be made by its manufacturer, is not guaranteed or endorsed by the publisher.
